# Trajectories in Care Among Veterans with Alzheimer’s Disease in the United States

**DOI:** 10.1002/alz.089903

**Published:** 2025-01-09

**Authors:** Natalia Palacios, Weiming Xia, Joel Reisman, Ying Wang, Peter J Morin, Dan Berlowitz, Amir Abbas Tahami Monfared, Quanwu Zhang, Byron J. Aguilar, Raymond Zhang

**Affiliations:** ^1^ Zuckerburg College of Health Sciences, University of Massachusetts Lowell, Lowell, MA USA; ^2^ Geriatric Research Education & Clinical Center, VA Bedford Healthcare System, Bedford, MA USA; ^3^ University of Massachusetts Lowell, Lowell, MA USA; ^4^ Center for Healthcare Organization & Implementation, VA Bedford Healthcare System, Bedford, MA USA; ^5^ Wentworth Institute of Technology, Boston, MA USA; ^6^ Boston University Chobanian & Avedisian School of Medicine, Boston, MA USA; ^7^ Eisai, Inc, Woodcliff Lake, NJ USA; ^8^ Alzheimer’s Disease & Brain Health, Eisai Inc., Nutley, NJ USA; ^9^ The Bedford VA Research Corporation, Inc., Bedford, MA USA

## Abstract

**Objective:**

Few studies have investigated trajectories in care among patients with Alzheimer’s Disease (AD), especially Veterans who experience a unique set of AD risk factors and challenges in access to AD immunotherapy in the Veterans Affairs Healthcare System (VAHS) of the United States.

**Methods:**

We analyzed trajectories in care based on electronic health records (EHR) among Veterans who were assessed for AD based on ICD‐10 coding within the VAHS between Oct 2015 and Jan 2024. Among Veterans with an ICD‐10 code for AD in the VAHS, we examined frequency distributions of clinical visits by physician specialty (primary care, neurologist, geriatrician, psychiatrist, and other specialties) where the initial AD ICD‐10 coding was recorded. Transition to the specialist care or visit to a dementia clinic was calculated during the year after the initial ICD‐10 coding for AD from the initial visit to a primary care physician. Distribution of the transition to specialist care was examined across non‐Hispanic Black, non‐Hispanic White, Hispanic, and other race/ethnicity groups.

**Results:**

A total of 110,836 Veterans were identified by ≥1 ICD‐10 code for AD with a subsample of 71,666 seen by a clinician with known specialty. Mean age was 80.7 years, 97.3% were male, 11.6% non‐Hispanic Black, 8.2% Hispanic, and 70.7% non‐Hispanic White. More than 41% of the initial AD coding was assigned in the primary care physician, 9% in neurologist, 8% geriatrician, and 12% psychiatrist. In the subsample of 71,666 Veterans within one year, 26.4% had a subsequent visit to a neurologist office or Dementia clinic. Among Veterans initially seen in primary care for AD, 31.7% non‐Hispanic Black (p<0.01) and 25.3% Hispanic (p = 0.22), relative to 23.9% non‐Hispanic White, were subsequently visited a neurologist or a dementia clinic.

**Conclusions:**

Over 40% more Veterans were initially evaluated for AD by a primary care clinician than by an AD specialist including neurologists, psychiatrists, and geriatricians. Non‐Hispanic Black Veterans were more likely to follow up with a neurologist or at a dementia clinic compared to Hispanic or non‐Hispanic White Veterans during the year after the initial AD assessment. The findings suggest a need for further enhancement of AD care trajectory.

